# Effects of music intervention on golf-specific skill performance of golfers under mental fatigue: Protocol for a randomized controlled trial

**DOI:** 10.1371/journal.pone.0337905

**Published:** 2025-12-04

**Authors:** Xiaoyang Pan, Kim Geok Soh, Wan Marzuki Wan Jaafar, He Sun, Guoyao Zhang

**Affiliations:** 1 Department of Sport Studies, Faculty of Education Studies, Universiti Putra Malaysia, Serdang, Selangor, Malaysia; 2 Department of Counsellor Education and Counselling Psychology, Faculty of Educational Studies, Universiti Putra Malaysia, Serdang, Selangor, Malaysia; 3 School of Physical Education, Henan University, Kaifeng, Henan, China; 4 Department of Physical Education, Faculty of Arts and Sciences, Chengdu College of University of Electronic Science and Technology, Chengdu, Sichuan, China; Southwest University, CHINA

## Abstract

Given that golf tournaments last approximately five hours without an intermission, mental fatigue has become a critical factor affecting performance. Although research has shown that music intervention can effectively alleviate mental fatigue, this method has yet to be applied in golf. Therefore, this study aims to evaluate the effects of music intervention on golf-specific skill performance under mental fatigue. This study adopts a randomized, controlled, partially-blind design, including three groups. The MF-Mu group undergoes a mental fatigue induction task followed by a music intervention. The MF-nMu group completes the same mental fatigue task but does not receive the music intervention. The CON group serves as the control and receives neither the mental fatigue task nor the music intervention. Controlled variables include immersion tendency, sport anxiety, attention, motivation, and perceived exertion. Primary outcome measures include mental fatigue, driving shot, iron shot, chipping shot, and putting performance. Performance measures include accuracy and shot quality. In this study protocol, the use of smartphones for music intervention enhances its portability, allowing the intervention to be initiated or terminated at any time, increasing its flexibility and acceptability. Moreover, briefly listening to music during non-striking moments complies with golf regulations, making this study highly practical. It provides an effective strategy for golfers to manage mental fatigue in competitive environments.

Trial registration

ClinicalTrials.gov NCT06952283

## Introduction

One of the biggest differences between golf and other Olympic sports is the complex playing environment and long duration of competitions. Specifically, golf courses incorporate various natural elements (such as undulating terrain, bunkers, water hazards, and vegetation) [1–3], which contribute to its strategic complexity [4–7]. Additionally, each round of golf takes around five hours without a halftime break, during which golfers need to maintain attention and cognitive effort for extended periods [8–12]. Long periods of cognitive tasks, emotional challenges, and stress can lead to mental fatigue [13–15]. Symptoms of mental fatigue include mental exhaustion, lack of energy, feelings of tiredness, decreased attention, and impaired cognitive and behavioral performance [16–18]. In sports, mental fatigue can damage athletes’ physical, psychological, tactical decision-making, and sport-specific skill performance [19–21]. Research on mental fatigue in basketball and soccer is relatively well-developed [22,23], with various effective strategies proposed to recover from mental fatigue and enhance performance [24]. However, research in the field of golf, particularly regarding mental fatigue recovery strategies, is still scarce [25,26]. Therefore, conducting research on mental fatigue recovery and performance enhancement for golf athletes is of significant importance.

Many studies have shown that the negative impact of mental fatigue on skill performance may be attributed to a reduction in dopamine transmission, which adversely affects executive functions [21,27–29]. Dopamine plays a central role in reward, motivation, cognition, and motor control in the brain [30], and is one of the most commonly proposed mechanisms related to mental fatigue [31,32]. This theory suggests that prolonged mental exertion may lead to the accumulation of the neuromodulator adenosine, which increases the perception of effort in subsequent activities [31–33]. Martin et al. [20] further expanded this hypothesis, stating that adenosine accumulation inhibits dopamine release in the anterior cingulate cortex, thereby reducing motivation or the willingness to exert effort. Consequently, some researchers have suggested that recovery strategies targeting dopamine system modulation, such as music and caffeine, may be effective for combating mental fatigue [18,33]. However, nutritional supplements like caffeine [34,35], carbohydrate mouth rinses [36,37], and creatine [38], while helpful in alleviating mental fatigue, have delayed effects, carry economic costs with frequent use, and may affect sleep quality (such as caffeine) or increase body weight (such as creatine), making them unsuitable for long-term use [39]. In contrast, finding alternative methods that produce immediate effects and are cost-effective for reducing mental fatigue is crucial. Therefore, non-invasive brain stimulation may be an effective strategy to address mental fatigue [39].

Music therapy, as a non-invasive and low-risk intervention, has already achieved significant results in the medical field [40–44]. A large body of research has demonstrated that music therapy can effectively reduce anxiety, improve depressive symptoms, and help patients alleviate physical pain [42,43,45]. Additionally, a systematic review of 139 studies (involving 3,599 participants) by Terry et al. [46] found that listening to music significantly improved participants’ positive emotional experiences, reduced subjective fatigue, and enhanced physical performance. Music intervention strategies have been successfully applied to basketball players, with studies showing that music relaxation can help basketball players quickly recover from states of self-exhaustion and maintain performance [47] However, music intervention strategies have not yet been applied to golfers [24,48].

Therefore, the aim of this study is to investigate the impact of music intervention on the golf-specific skill performance of Chinese golfers under mental fatigue. Through experimental research, the study will analyze whether music intervention can effectively mitigate the negative effects of mental fatigue and enhance the performance of golfers’ specialized skills. Additionally, the study will examine the differential impact of mental fatigue and music intervention on various skills. The findings will not only provide practical strategies for golf coaches and athletes to recover from mental fatigue and improve competitive performance under such conditions, but also offer valuable insights for other sports that require high concentration and resilience under pressure.

## Methods and design

### Status and timeline

**Fig 1:**
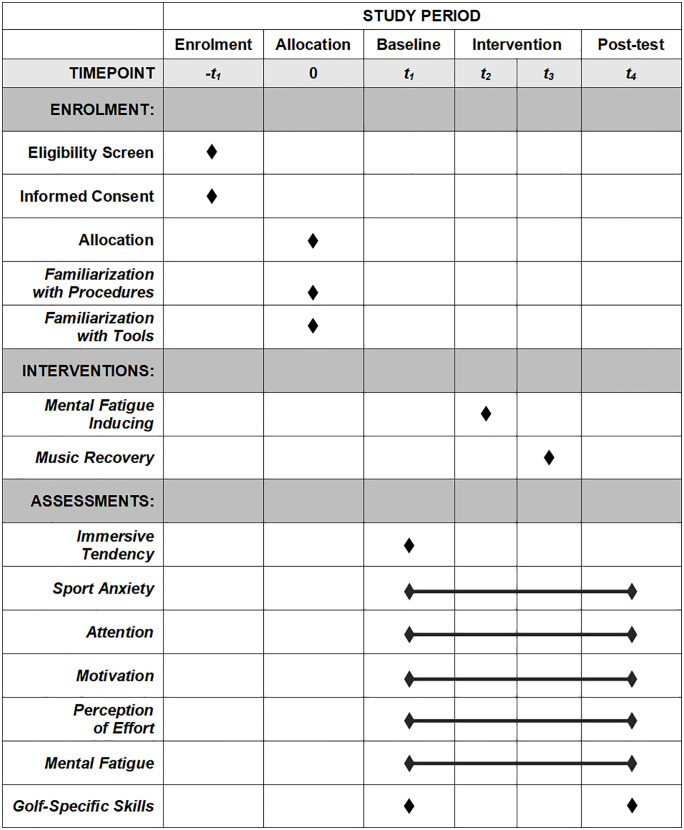
SPIRIT schedule of enrollment, interventions, and assessments.

This study protocol has been approved by the Ethics Committee of Henan University, China (Approval No.: HUSOM2025−066) and strictly adheres to the ethical principles of the Declaration of Helsinki. Additionally, this trial was registered on ClinicalTrials.gov in April 2025 under the identifier NCT06952283. Fig 1 presents an overview of the study timeline. At present, neither participant recruitment nor data collection has commenced. The study is scheduled to begin participant recruitment on October 1, 2025, with recruitment expected to be completed by October 31, 2025. Data collection is anticipated to be completed by November 30, 2025, and the goal is to complete data analysis and obtain study results by December 31, 2025. Subsequently, we plan to disseminate the results to the broader public through publications in reputable peer-reviewed scientific journals and presentations at academic conferences

### Participants

Following recommendations from Lakens [49] and Aberson [50], powering for interaction effects is considered essential in behavioral intervention trials where the primary interest lies in differential changes across conditions. Accordingly, the sample size calculation is based on the statistical power required to detect a time × group interaction effect, estimated using the “ANOVA: Repeated measures, within–between interaction” model in G*Power 3.1. Assuming a medium effect size f = 0.25 (Cohen’s standard), α = 0.05, power = 0.95, three groups, two measurement time points, and an intra-class correlation (ICC) of 0.5, the estimated result shows that the study requires at least 66 participants (22 per group) to ensure sufficient power for detecting the interaction effect. Considering a potential dropout rate of 15% [51], a total of 76 golfers will be recruited. If practical resource constraints result in a slightly smaller sample size, the study will report effect sizes and Bayes factors to supplement the interpretation of statistical significance and cautiously delimit the generalizability of conclusions to ensure the scientific rigor and interpretability of the results.

Participants in this study will be selected from the National Colleges and Universities Golf Championship (NCUGC) in China. The research team will collaborate with the head coaches of the participating teams to determine the universities willing to participate. Subsequently, the head coaches and team physicians will conduct an initial screening of players based on the inclusion criteria set for this study (Table 1). The research team will then verify the eligibility of the selected participants before the experiment begins.

**Table 1:** Participant selection criteria.

Inclusion criteria	Exclusion criteria
Aged 18–24 years old	Color blindness
Golfers of the NCUGC	Hearing loss
Least 3 years of specialized golf training	Insomnia
Least 5 trainings per week	Experiencing any physical injury
	Experiencing mental health issues
	Receiving any medication

To ensure the safety of all participants, a team physician will be present throughout the experiment to provide immediate medical support and guidance in case of discomfort or injury. All participants are covered by accident and medical insurance provided by their universities. Before the experiment begins, the research team will provide participants with a detailed explanation of the study’s objectives, procedures, and potential risks, and obtain written informed consent.

### Experimental design

**Fig 2:**
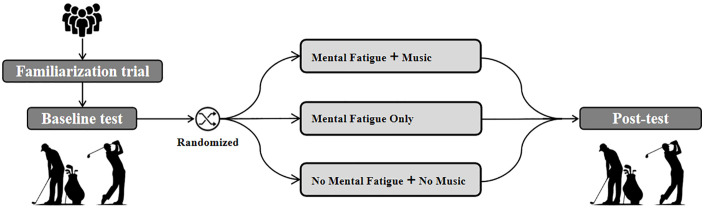
Overview of the experimental design.

This study protocol is based on the SPIRIT 2013 clinical trial protocol guidelines [52,53] (S1-S3 File) and follows a randomized, controlled, partially-blind design. This study will be conducted during a specially designed summer golf training camp, with all experimental procedures carried out within the camp period. During this time, all participants will follow the same diet, daily schedule, training, and competition routines to minimize dropout and external interference. The study consists of three main phases (Fig 2): familiarization with the experimental procedure, baseline testing, and the formal experiment. During the formal experiment, participants will be randomly assigned to one of the following three groups: (MF-Mu Group) Participants undergo a mental fatigue induction task followed by a music intervention; (MF-nMu Group) Participants undergo a mental fatigue induction task but do not receive a music intervention; (CON Group) Participants neither undergo a mental fatigue induction task nor receive a music intervention.

Randomization of group allocation and participant order will be conducted using a random number table generator (www.randomizer.org), ensuring that no one can predict the group assignments before randomization. Participants will not be informed of the true purpose of the study; instead, they will be told that the study is part of a summer golf training camp and that they should perform their best during the skill testing phase. During the intervention, dedicated assistants will be responsible for inducing mental fatigue and administering the music intervention, but they will not be informed of the specific research objectives. During the golf skill testing and performance recording phase, testing assistants will be responsible only for recording test results and will not be aware of the participants’ group assignments. Data analysis will be conducted by researchers who only have access to anonymized data. Group assignment information will only be disclosed to the on-site medical team in case of participant health concerns. This design is conducive to blinding control. [54–56].

The total intervention duration is 45 minutes, consisting of 30 minutes of mental fatigue induction and 15 minutes of music intervention. Participants not undergoing mental fatigue induction will read some magazine leisurely, a common control in mental fatigue trials [57–59]. Those not receiving the music intervention will sit quietly and rest, a common control in music intervention trials [60–62], to control external factors and maintain consistent conditions. Immediately after the intervention, participants will complete golf skill testing, with the testing site located within a 2-minute walking distance from the intervention area. Performance outcomes include driving performance, iron shot performance, chipping performance, and putting performance. Golf skill test data will be collected on-site by assistant researchers and verified and signed off by a national-level referee to ensure validity.

### Intervention protocols

The experiment will be conducted at the university’s golf training facility under the supervision of professional members of the research team, including the university team’s head coach, team doctor, and a national-level golf referee. Data will be collected before and after the intervention, covering questionnaire assessments and golf skill tests. The baseline test and the formal test will be one week apart, and the experiment will take place on the same day of the week and at the same time (9:00–11:00 AM) to ensure sufficient recovery time and minimize the interference of residual effects [55,63,64].

During the mental fatigue induction phase, a 30-minute Stroop task will artificially induce mental fatigue, simulating the mental fatigue state that athletes experience during competitions or training sessions [65]. The Stroop task is a classic cognitive control task widely used for inducing mental fatigue. Research in sports science has shown that a 30-minute Stroop task significantly increases subjective fatigue and reduces sports performance [21,24]. This study will use a smartphone-based version of the Stroop task, where colour words (such as “red” and “blue”) are displayed continuously, but the font colour does not match the word’s meaning. Participants must ignore the word’s meaning and respond only based on the font colour. The Stroop task requires sustained attention, conflict resolution, and cognitive inhibition, leading to cognitive resource depletion and mental fatigue accumulation. During the experiment, all participants will use their smartphones (with brightness and touch sensitivity standardised) and wear earphones to minimise external distractions. Participants in the MF-Mu and MF-nMu groups will undergo mental fatigue induction, while those in the CON group will only read a golf magazine in a relaxed manner.

During the music intervention phase, participants will listen to 15 minutes of classical music to facilitate mental fatigue recovery. Research has shown that short-term listening to classical music can effectively alleviate mental fatigue [60,66–68]. Among classical music, Mozart’s compositions are particularly effective in cognitive function recovery, attention enhancement, emotional regulation, and mental fatigue relief due to their bright and optimistic style, pure and elegant melodies, and harmonious rhythms [69–71]. Therefore, This study selects Mozart’s Eine Kleine Nachtmusik as the intervention music [72], a piece that has been applied in previous mental fatigue recovery studies and has been validated as an effective intervention [66]. The intervention duration is set at 15 minutes, as multiple studies have confirmed this duration to be sufficient to produce recovery effects without causing additional cognitive load or auditory fatigue [66,73,74]. Based on research in the field of music therapy, the intervention volume should be maintained between 50–60 dBA [71,75–77]. The music will be played through a smartphone. Considering that the maximum volume of a smartphone is approximately 105–113 dBA [78], the standardized intervention volume was set at 50% of the maximum (52.5–56.5 dBA), with participants allowed to make slight adjustments to ensure listening comfort. Participants in the MF-Mu group will wear earphones and listen to the music for 15 minutes, while those in the MF-nMu and CON groups will wear earphones in the same environment but without audio, simply sitting at rest for 15 minutes. This experimental design effectively controls for non-musical factors, ensuring that the observed effects can be solely attributed to the music intervention.

The mental fatigue induction and music intervention will be conducted in the same classroom, with the room temperature maintained at 24°C, soft lighting, and no background noise. This study has chosen smartphones instead of computers as the experimental devices because listening to music via smartphones during non-hitting intervals in official golf competitions complies with golf regulations. Thus, using smartphones enhances the real-world applicability of the study’s conclusions in actual competition settings.

### Measures

This experiment measures multiple variables to comprehensively assess the effects of experimental conditions, including mental fatigue and music intervention, on golf skill performance. The measured variables include mental fatigue, immersion tendency, sport anxiety, attention, motivation, perceived exertion, and golf skill performance (Table 2).

**Table 2:** Summary of Instruments.

Item	Variable	Instrument	Inventor & Citation	Scale
Independent variable	Mental Fatigue	VAS-MF	Hayes & Patterson, 1921	100 mm scale
Control variables	Immersive Tendency	ITQ	Witmer & Singer, 1998	7-point Liker scale
	Sport Anxiety	SAS-2	Smith et al., 2006	4-point Liker scale
	Attention	SAS-2	Smith et al., 2006	4-point Liker scale
	Motivation	VAS-Mo	Hayes & Patterson, 1921	100 mm scale
	Rating Perception of Effort	Borg RPE	Borg, 1982	15-level scale
Dependent variables	Driving performance	Driving Test	China Golf Association, 2019	10 shots
	Iron performance	150Y Iron Test	China Golf Association, 2019	10 shots
	Chipping performance	30Y Chipping Test	China Golf Association, 2019	10 shots
	Putting performance	Putting Test	China Golf Association, 2019	10 shots

VAS: Visual Analogue Scale; MF: mental fatigue; Mo: motivation; ITQ: Immersive Tendencies Questionnaire; SAS-2: Sport Anxiety Scale-2; Borg RPE: Borg rating of perceived exertion scale; Y: Yard

### Visual analog scale

The Visual Analog Scale (VAS) is used to assess participants’ subjective perception of mental fatigue and task motivation levels [79]. The scoring is based on a 100 mm linear scale, where participants mark a point between 0 (none at all) and 100 (extremely intense) to describe their current level of mental fatigue or motivation.

### Immersive tendency questionnaire

The Immersive Tendency Questionnaire (ITQ) is used to assess participants’ degree of immersion in the virtual environment (music) [80]. This questionnaire consists of 18 items categorized into four factors, including immersive tendency, individual alertness, physical state, and attention-shifting ability. Each item is rated on a 7-point Likert scale, ranging from 1 (strongly disagree) to 7 (strongly agree).

### Sport anxiety scale-2

The Sport Anxiety Scale-2 (SAS-2) assesses participants’ trait sport anxiety levels [81]. This scale consists of 15 items categorized into three factors: somatic anxiety, worry, and concentration disruption. Each item is rated on a 4-point Likert scale, ranging from 1 (not at all) to 4 (very much so).

### Borg rating of perceived exertion

The Borg Rating of Perceived Exertion (Borg RPE) is used to assess individuals’ subjective effort levels [82]. This scale employs a 15-level scoring system, ranging from 6 (extremely easy) to 20 (extremely strenuous), to reflect athletes’ subjective perception of workload intensity during testing, or competition.

### Golf-specific skill tests

**Fig 3:**
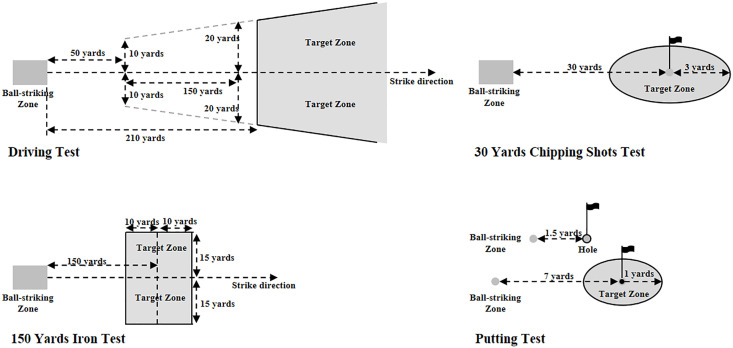
Schematic of site setup and valid zone.

The golf-specific skill tests follow the “Golf Skill Level Standards and Testing Methods” established by the China Golf Association [83]. The skill tests include four core skill assessments: driving performance, 150 yards iron shot performance, 30 yards chipping performance, putting performance (Fig 3).

The test venue is set up under the supervision of national-level golf referees from the China Golf Association and certified examiners of the Golf Skill Level Standards and Testing Methods to ensure standardized test conditions. During the testing process, these officials will oversee the entire procedure and make rulings on any disputes related to rules and scores. Practice balls and tees used in the test are provided by the research team. The practice balls are two-piece balls that meet golf equipment regulations, with consistent models and conditions maintained. Participants use their own clubs and gloves to prevent performance variations due to unfamiliar equipment. However, all equipment must comply with the relevant regulations of the Rules of Golf Equipment [84]. The golf referees and examiners on-site will strictly inspect participants’ personal equipment to ensure compliance with regulations. Each skill test requires 10 shots, and the final score is determined by the number of shots that land within the designated valid area. To ensure optimal performance and prevent injuries, all participants will complete a standardized 10-minute warm-up routine led by the head coach before taking the golf-specific skill tests [85,86].

### Data management

All participants signed informed consent forms and voluntarily participated in the experiment. Data management in this study strictly adheres to the General Data Protection Regulation of April 27, 2016, ensuring data confidentiality. The collected data will be used solely for academic research and will not be utilized for any commercial purposes. Participation in this study implies that subjects agree to allow researchers to collect their data and publish the research results in scientific journals. Participants retain the right to access their collected data and may request corrections if errors are found. Additionally, they have the right to withdraw from the study at any time without providing any reason.

All data will be recorded and entered by members of the research team. Psychological measurement data and skill performance data will be documented using paper-based questionnaires and forms. To ensure participant privacy and data security, paper-based data will be entered into the researchers’ electronic database within 24 hours and immediately destroyed. All data will be anonymized using coded identifiers to protect participant identities, with data access restricted to research team members. During data collection, the research team will implement quality control measures, including data integrity checks, cross-checking, and error correction. All electronic data will be regularly backed up, with backup files stored in a cloud storage system. Research findings and data analysis will be made publicly available through peer-reviewed journals or conference presentations.

### Statistical analysis

All statistical tests will be conducted at a significance level of α = 0.05, with effect sizes measured using partial eta squared (η²p). The statistical power is set at 0.80, and all analyses will be performed using IBM SPSS Statistics 27.0. This study will adopt a 2 (Time: pre-test vs. post-test) × 3 (Group: mental fatigue–music intervention group, mental fatigue–non-music group, control group) mixed-design repeated measures ANOVA to examine the effects of mental fatigue and music intervention on golf-specific skill performance. The core hypothesis of the study is the time × group interaction effect, which aims to determine the performance differences across groups before and after the intervention, thereby verifying the potential value of music intervention under mental fatigue. Time main effects and group main effects will serve as secondary analysis objectives.

Prior to the main analysis, a one-way ANOVA will be conducted to compare baseline skill performance across the three groups to assess initial group equivalence and ensure the validity of subsequent comparisons. In the main effects analysis, mixed-design repeated measures ANOVA will be used to test the time main effect (whether overall skill performance changes over time), group main effect (whether there are overall differences among the three groups), and the time × group interaction effect (whether skill performance changes differ among the groups). If the assumption of sphericity is violated, the Greenhouse-Geisser or Huynh-Feldt correction will be applied to adjust the degrees of freedom. If the interaction effect is significant, simple effects analysis will be conducted to clarify the performance trends before and after the intervention within each group and to compare differences between groups at each time point.

To control the accumulation of Type I error due to multiple comparisons, all post hoc group comparisons will apply Bonferroni correction. If the study involves multiple primary outcomes or multiple independent hypothesis tests, the Holm-Bonferroni method will be further applied to control the family-wise error rate. In addition, the study will predefine a single primary outcome—ball-shooting accuracy score—while other technical indicators such as shot quality and reaction time will be treated as exploratory outcomes, serving to support and interpret the results but not as primary hypothesis testing targets.

All statistical analyses will report effect size indicators. For significant results, partial eta squared (η²p) will be reported as the effect size for ANOVA, and Cohen’s d will be calculated for between-group differences to reflect the practical significance of the effects. In addition to traditional significance testing, Bayesian factor analysis (Bayes Factor Analysis) will be conducted, and BF₁₀ values will be reported to provide relative evidence strength between the null and alternative hypotheses, enhancing the multidimensional reliability of the interpretations.

This study is designed as a closed golf summer training camp, and all experimental measurements will be conducted within a unified time window. This centrally managed experimental environment helps to minimize the risk of dropouts or missing data due to personal factors (such as going out, absences), but the risk of missing data in repeated measures design still needs to be considered. Missing data will be handled using mixed-effects models combined with maximum likelihood estimation (ML) to retain all available data and obtain robust and unbiased effect estimates. When the proportion of missing data is high (such as more than 5%), multiple imputation will be adopted, and sensitivity analyses will be conducted to explore the impact of the missing data mechanism on study conclusions, thereby enhancing the credibility of the inferences.

## Discussion

Golf rounds last five hours with no halftime break, and golfers’ sustained attention and cognitive effort can lead to mental fatigue [13–15]. Mental fatigue is almost inevitable in actual competitions [27,87,88], making the development of effective psychological recovery strategies to alleviate mental fatigue and optimize athletic performance an important task in current sports science research [33,89].

This study is the first to focus on the impact of music intervention on golf skill performance under mental fatigue conditions, filling a gap in this field of research. The “mental fatigue induction + music intervention + golf skill testing” experimental design provides a multidimensional research perspective. A major highlight of this study is the use of smartphones as the music intervention device. Smartphones are portable, enabling quick initiation or termination of the intervention without disrupting the rhythm of the game, enhancing the flexibility and acceptability of the intervention. Additionally, using a smartphone to listen to music during non-swing times aligns with the rules of golf [84], making this intervention method practically feasible. The study results could provide guidance for golfers, coaches, and sports psychologists to optimize mental fatigue management and competition preparation strategies. If the results confirm its effectiveness, the intervention could be applied in golf and other competitive sports training and competitions.

An additional strength of the present study lies in its implementation within a specially organized summer golf training camp. This closed and centralized management setting is expected to significantly reduce the likelihood of participant dropout due to personal reasons and minimize potential external confounders that could affect experimental outcomes. As a result, this design enhances both data completeness and the internal validity of the study. Such a controlled training camp model offers valuable methodological advantages that extend beyond golf research. It may serve as a useful framework for experimental studies in other sports disciplines, particularly when aiming to investigate the effects of cognitive, psychological, or physiological interventions under ecologically valid conditions. Future sports science research may benefit from adopting similar closed-environment approaches to balance experimental control with real-world relevance.

## Supporting information

S1 File SPIRIT 2013 checklist. (PDF)

S2 File Respondent’s information sheet and informed consent. (PDF)

S3 File Approved experimental study protocol. (PDF)
