# Supplementary material for: Effects of music intervention on golf-specific skill performance of golfers under mental fatigue: Protocol for a randomized controlled trial
Source: PLoS One. 2025 Dec 4;20(12):e0337905. doi: 10.1371/journal.pone.0337905 (PMC12677511; doi:10.1371/journal.pone.0337905)
Supplement: S2 File — (PDF) [file pone.0337905.s002.pdf]

S2. Respondent's information sheet and informed consent.

RESPONDENT'S INFORMATION SHEET AND INFORMED CONSENT FORM

Dear Participant,

Thank you for responding to our invitation and considering participation in this study. Please read the following information carefully and ensure you fully understand it before deciding whether to participate. If you have any questions, feel free to consult the research team.

1. STUDY TITLE
Effects of Music Intervention on Golf-Specific Skill Performance of Chinese Golfers Under Mental Fatigue

2. INTRODUCTION
This study is a randomized controlled trial in the field of sports science, aiming to investigate the effects of music intervention on golf-specific skill performance under mental fatigue. Previous research suggests that prolonged cognitive activity can lead to mental fatigue, which is characterized by exhaustion, decreased energy levels, increased feelings of fatigue, and reduced concentration. These factors may negatively affect athletic performance. Golf is a sport with complex environmental conditions and long competition durations, making mental fatigue almost unavoidable. This study seeks to scientifically analyze whether music intervention can effectively alleviate the negative effects of mental fatigue and provide data to support mental fatigue management for golfers in training and competition. Your participation will provide valuable data for this research and contribute to the advancement of this field.

3. WHAT WILL YOU HAVE TO DO?
If you agree to participate in this study, we will schedule three sessions (total 3 weeks). In first session, you will learn about the experimental procedures and familiarize yourself with the relevant questionnaires. In second session, you will undergo baseline testing to assess your initial condition. In third session, you will be randomly assigned to one of three groups: the mental fatigue with music intervention group, the only mental fatigue group, or the control group (no mental fatigue and no music). Each group has an equal probability of 33.3%. Each session will be spaced one week apart to ensure sufficient recovery time. This study does not involve any invasive procedures. All experiments will be conducted under the supervision of experienced researchers, following safety regulations to ensure your comfort and well-being.

4. WHO SHOULD NOT PARTICIPATE IN THE STUDY?
We need about 48 for the participants. If you do not reach the competitive level of college national competitions, if you are experiencing serious physical or mental health problems, if you have received relevant medication in the past 6 months, if you have adverse reactions to music or specific sounds (such as anxiety, headaches, etc.), then you are not suitable for participating in this study. In order to ensure the reliability of the research data, you need to comply with the following requirements, otherwise you are not suitable for participating in the study:

- Avoid strenuous exercise or long-term cognitive activities (except daily learning and training), and ensure adequate sleep and avoid staying up late;
- Avoid consuming high-energy foods, alcohol or caffeine (except normal drinking water), and ensure that you eat in the athlete restaurant.

5. WHAT WILL BE THE BENEFITS OF THE STUDY:
(a) TO YOU AS THE SUBJECT?
Course credit will be given for this study. During this study, you will undergo a sports psychological assessment and a golf skill performance test, which will help you better understand your mental state and

competitive level. Additionally, by participating in this study, you will have the opportunity to access the latest research findings on music intervention for mental fatigue management. These findings may provide a scientific basis for optimizing your mental fatigue management strategies in training and competition.

(b) TO THE INVESTIGATOR?

Your participation will provide valuable scientific data on the effects of music intervention on golf-specific skill performance, helping researchers gain deeper insights into the mechanisms of mental fatigue and its impact on golfers' performance. This will contribute to the development of evidence-based intervention strategies for golfers and coaches to optimize performance and serve as foundational data for future research in this field.

6. WHAT ARE THE POSSIBLE RISKS?

This study may cause mild discomfort, primarily due to the mental fatigue induction process. After completing cognitive tasks, you may experience reduced energy levels, increased fatigue, or decreased concentration. However, we will provide adequate rest and psychological support and take necessary measures to ensure your safety and well-being. This experiment is covered under the university insurance policy. You may withdraw from this study at any time without providing any reason. Withdrawal will have no consequences and will not be recorded.

7. WILL THE INFORMATION THAT YOU PROVIDE AND YOUR IDENTITY REMAIN CONFIDENTIAL?

You have the right to access your own measurement data. Your personal information and experimental data will be strictly confidential and used only for research purposes. Your identity will not be disclosed, and all data will be processed anonymously. When research results are published, your personal information will not be revealed. Additionally, your data will be stored in a secure environment, accessible only to authorized research team members, and will not be shared with any third party without your explicit consent.

8. WHO SHOULD YOU CONTACT IF YOU HAVE ADDITIONAL QUESTIONS DURING THE COURSE OF THE RESEARCH?

During the experiment, research staff will be present to assist you at all times. If you have any questions, feel free to speak with the research personnel accompanying you.
If you need further information about the study or have any concerns, you can contact:
Name:
Institution:
Phone:
Email:

If you have any questions about your rights as a participant in this study, please contact:
The Secretariat, JKEUPM, at email address **jkeupm@upm.edu.my**

Please initial here if you have read and understood the contents of this page________

9. CONSENT

My name is .... gender .... age .... dominant hand ....
Identity Card No .... address ....
I * am / am not a university golfer at the national competition level, and my golf handicap is .... I have been training in golf for .... years, with training sessions .... times per week. I voluntarily agree to participate in the aforementioned research * (questionnaire survey / experimental procedure / skill testing).

I have been informed about the nature of the research in terms of methodology, possible adverse effects and complications (as written in the Respondent's Information Sheet). I understand that I have the right to withdraw from this research at any time without giving any reason whatsoever. I also understand that this study is confidential and all information provided with regard to my identity will remain private and confidential.

I* wish / do not wish to know the results related to my participation in the research.

I* agree/do not agree that my measurement data to be used in any form of publication or presentation.

* *delete where necessary*

Signature …….…………………………
(Respondent)

Date :……………………………………..

Signature …….…………………………
(Witness)

Name :……………………………………..

I/C No. :……………………………………..

I confirm that I have explained to the respondent the nature and purpose of the above-mentioned research.

Date …….…………………………

Signature …….…………………………
(Researcher)

受访者的信息表及知情同意书

尊敬的参与者，

感谢您回应我们的邀请，并考虑加入本研究。请您仔细阅读以下信息，并在充分理解后决定是否同意参与。如果您有任何疑问，请随时咨询研究人员。

1. 研究标题

《音乐干预对中国高尔夫球员在精神疲劳下的高尔夫专项技能表现的影响》

2. 简介

本研究属于一项运动科学领域的随机对照实验，旨在探讨音乐干预对精神疲劳下高尔夫专项技能表现的影响。已有研究表明，长时间的认知活动可能导致精神疲劳，其典型表现包括精神枯竭、能量不足、疲惫感增加以及专注力下降，而这些因素可能会损害运动员的竞技表现。高尔夫是一项环境复杂、竞赛时间漫长的运动，因此，精神疲劳几乎无法避免。本研究希望通过科学实验，分析音乐干预是否能够有效缓解精神疲劳带来的负面影响，并为未来高尔夫运动员在训练和比赛中的精神疲劳管理提供数据支持。您的参与将为本研究提供宝贵的数据，并有助于推动该领域的发展。

3. 您需要做什么？

如果您同意参与本研究，我们将有三次会面（共 3 周）。第一次会面中，您将了解实验程序，熟悉相关问卷。第二次会面中，您将进行基线测试，以评估您的基础状态。第三次会面中，您将被随机分配到三组：精神疲劳干预组、精神疲劳组，或对照组（无精神疲劳且无音乐）。被随机分配到每组的概率均为 33.3%。每次会面间隔一周，以确保您有充分的恢复时间。本研究不会涉及任何侵入性程序，所有实验均由经验丰富的研究人员指导，并在符合安全规定的情况下进行，以确保您的安全与舒适。您可以随时退出实验，退出不会对您有任何影响。

4. 谁不能参加本研究？

我们需要约 48 位参与者。如果您未达到大学生全国比赛的竞技水平，如果您正在经历严重的身体或精神健康问题，如果您过去 6 个月内接受过相关药物治疗，如果您对音乐或特定声音存在不良反应（如焦虑、头痛等），那么您不适合参与本研究。为了确保研究数据的可靠性，您需要遵守以下要求，否则不适合参加研究：

- 避免剧烈运动或长时间的认知活动（日常学习和训练除外），并确保充足睡眠，避免熬夜；
- 避免摄入高能量食物、酒精或咖啡因（正常饮水除外），并确保在运动员餐厅统一就餐。

5. 参加本研究有什么好处:

a) 对您作为参与者的好处

本研究将赋予课程学分。在本研究过程中，您将接受运动心理状态评估和高尔夫技能水平测试，帮助您更

好地了解自己的运动心理状况和竞技水平。此外，通过参与本研究，您将有机会获取关于音乐干预在精神疲劳管理中的最新研究成果，这些研究结果可能为您在比赛和训练中优化精神疲劳管理策略提供科学依据。

b) 对研究人员的好处

您的参与将为我们提供关于音乐干预对高尔夫专项技能表现的科学数据，帮助研究人员更深入地理解精神疲劳的机制及其对高尔夫运动员表现的影响。这将为高尔夫运动员及教练提供基于科学证据的干预方案，以优化运动表现，并为未来相关研究提供基础数据支持。

6. 可能存在的风险是什么?

本研究可能会引起轻微的不适，主要来源于精神疲劳诱发过程。在完成认知任务后，您可能会感到精神不足、疲惫感增加或专注力下降。然而，我们将提供足够的休息和心理支持，并采取措施确保您的安全。本实验属于大学保险覆盖范围内。您可以随时退出本研究，无需提供任何理由。退出不会带来任何不良后果，也不会被记录。

7. 您所提供的信息和您的身份是否会保密?

您有权访问自己的测量数据。您的个人信息和所有实验数据将严格保密，仅用于本研究的目的。您的身份不会被公开，所有数据均匿名处理，在研究结果发布时，您的个人信息不会被披露。此外，您的数据将存储在安全的环境中，仅研究团队授权成员可访问，且未经您的明确同意，数据不会被提供给任何第三方。

8. 如果在研究过程中遇到其他问题，应与谁联系?

实验过程中，研究人员将全程陪同，如有任何问题，您可以随时与身边的研究人员沟通。
如果您需要进一步了解研究相关信息或有任何疑问，可以联系下方研究者：
姓名:
机构:
电话:
邮箱:

如果您对作为本研究参与者的权利有任何疑问，请通过以下方式联系：UPM 伦理委员会秘书处 (JKEUPM)
电子邮箱：jkeupm@upm.edu.my

如果您已阅读并理解 1-2 页的内容，请在此处签名 ____________

9. 知情同意书

我叫……………………………………………………………性别………………年龄………………惯用手………………

证件号码…………………………………………………………地址…………………………………………………………。

我*（是 / 不是）国家级水平的大学高尔夫球员，个人差点指数为……………。我已经进行高尔夫专项训练……………年，每周训练……………次。特此自愿同意参加上述研究 *（问卷调查 / 实验操作 / 技能测试）。

我已经被告知研究的性质，可能的不利影响和并发症（如受访者信息表中所述）。我知道我有权在任何时候退出这项研究，而不用给出任何理由。我也明白这项研究是保密的，提供的所有有关我身份的信息将保密。

我*（感兴趣 / 不感兴趣）知晓涉及我的研究结果。

我*（同意 / 不同意）允许我的测量数据用于任何形式的出版或展示。

* *请划去不相关选项*

签名 ……..……………………………… *(受访者)*

日期 ……………………………….……..

签名 ……..……………………………… *(见证人)*

姓名 ………………………………….…..

证件号码 ……………………………..……..

本人确认，我已向受访者解释了上述研究的性质和目的。

日期 ……..………………………………

签名 ……..……………………………… *(研究人员)*
