# Supplementary material for: Effects of music intervention on golf-specific skill performance of golfers under mental fatigue: Protocol for a randomized controlled trial
Source: PLoS One. 2025 Dec 4;20(12):e0337905. doi: 10.1371/journal.pone.0337905 (PMC12677511; doi:10.1371/journal.pone.0337905)
Supplement: S3 File — (PDF) [file pone.0337905.s003.pdf]

S3. Approved experimental study protocol.

Experimental Trial Research Protocol (English Translation)

Overview of Human Experiment Protocol：

Optimizing Sport-Specific Performance from the Perspective of Mental Fatigue

Study Title：

Effect of Music Intervention on Golf-Specific Skill Performance Under Mental Fatigue

Overview：

This study is part of a series of studies. Participants are Chinese golfers. The study **does not involve** any clinical trials of drugs or compounds, clinical trials of medical devices, trials of supplements, human genetic studies, or the collection of human tissue samples, bodily fluids, or physiological measurements. The experimental protocol includes only questionnaires, cognitive tasks, and sport-specific skill tests.

Content includes study objectives, methods, sample size, and expected outcomes：

1. Research Background：
One of the biggest differences between golf and other Olympic sports is the complex playing environment and long duration of competitions. Specifically, golf courses incorporate various natural elements (such as undulating terrain, bunkers, water hazards, and vegetation) (Graves & Cornish, 1998; Farrally et al., 2003; Fouillouze et al., 2023), which contribute to its strategic complexity (Broadie, 2012; Pilgrim et al., 2016; Pilgrim et al., 2018; Brown & Fry, 2022). Additionally, each round of golf takes around five hours without a halftime break, during which golfers need to maintain attention and cognitive effort for extended periods (Giacobbi et al., 2004; Cotterill et al., 2010; Campbell & Moran, 2014; Hasegawa et al., 2021; Shaw et al., 2021). Long periods of cognitive tasks, emotional challenges, and stress can lead to mental fatigue (Mizuno et al., 2011; Tanaka et al., 2014; Finsterer & Mahjoub, 2014). Symptoms of mental fatigue include mental exhaustion, lack of energy, feelings of tiredness, decreased attention, and impaired cognitive and behavioral performance (Qi et al., 2019; Habay et al., 2021; Proost et al., 2022). In sports, mental fatigue can damage athletes' physical, psychological, tactical decision-making, and sport-specific skill performance (Van Cutsem et al., 2017; Martin et al., 2018; Sun et al., 2021).
2. Problem Statement：

Research on mental fatigue in basketball and soccer is relatively well-developed (Sun et al., 2022a; Cao et al., 2022), with various effective strategies proposed to recover from mental fatigue and enhance performance (Pan et al., 2024). However, research in the field of golf, particularly regarding mental fatigue recovery strategies, is still scarce (Pan et al., 2025). Therefore, conducting research on mental fatigue recovery and performance enhancement for golf athletes is of significant importance.

Music therapy, as a non-invasive and low-risk intervention, has already achieved significant results in the medical field (Magee, 2005; Ghetti, 2012; Cosmo et al., 2022; Lorek et al., 2023; Du et al., 2024). Music intervention strategies have been successfully applied to basketball players, with studies showing that music relaxation can help basketball players quickly recover from states of self-exhaustion and maintain performance (Englert & Bertrams, 2016). However, music intervention strategies have not yet been applied to golfers (Pan et al., 2024; Ding et al., 2025).

3. Study Objectives：

The overall objective of this study is to investigate the effect of music intervention on sport-specific skill performance among Chinese golf athletes under mental fatigue. It includes two specific aims:

(1) To explore the negative impact of mental fatigue on sport-specific skill performance in Chinese golf athletes;

(2) To explore the recovery effect of music intervention on sport-specific skill performance in Chinese golf athletes experiencing mental fatigue.

4. Methods：

4.1 Experimental Design：

This study protocol is based on the SPIRIT 2013 clinical trial protocol guidelines (Chan et al., 2013a; Chan et al., 2013b) and follows a randomized, controlled, partially-blind design. The study consists of three main phases (**Figure 1**): familiarization with the experimental procedure, baseline testing, and the formal experiment. During the formal experiment, participants will be randomly assigned to one of the following three groups: (MF-Mu Group) Participants undergo a mental fatigue induction task followed by a music intervention; (MF-nMu Group) Participants undergo a mental fatigue induction task but do not receive a music intervention; (CON Group) Participants neither undergo a mental fatigue induction task nor receive a music intervention.

Figure 1 Overview of the experimental design.

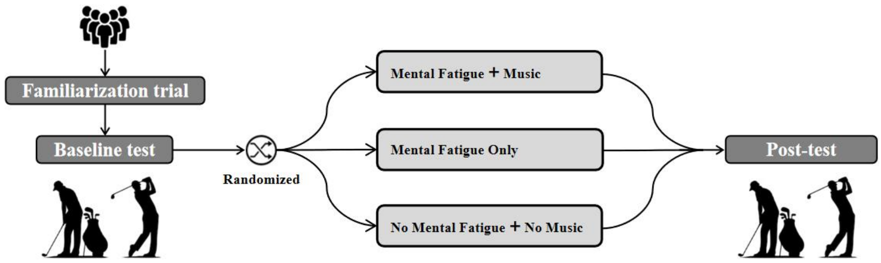

Randomization of group allocation and participant order will be conducted using a random number table generator (www.randomizer.org), ensuring that no one can predict the group assignments before randomization. Participants will not be informed of the true purpose of the study; instead, they will be told that the study is part of a quarterly training program and that they should perform their best during the skill testing phase. During the intervention, dedicated assistants will be responsible for inducing mental fatigue and administering the music intervention, but they will not be informed of the specific research objectives. During the golf skill testing and performance recording phase, testing assistants will be responsible only for recording test results and will not be aware of the participants' group assignments. Data analysis will be conducted by researchers who only have access to anonymized data. Group assignment information will only be disclosed to the on-site medical team in case of participant health concerns. This design is conducive to blinding control. (Angius et al., 2022; Arenales Arauz et al., 2024; Han et al., 2024).

The total intervention duration is 45 minutes, consisting of 30 minutes of mental fatigue induction and 15 minutes of music intervention. Participants not undergoing mental fatigue induction will read some magazine leisurely, a common control in mental fatigue trials (Smith et al., 2016; Veness et al., 2017; Davidow et al., 2023). Those not receiving the music intervention will sit quietly and rest, a common control in music intervention trials (Baghurst et al., 2014; Wang et al., 2020; Wang et al., 2022), to control external factors and maintain consistent conditions. Immediately after the intervention, participants will complete golf skill testing, with the testing site located within a 2-minute walking distance from the intervention area. Performance outcomes include driving performance, iron shot performance, chipping performance, and putting performance. Golf skill test data will be collected on-site by assistant researchers and verified and signed off by a national-level referee to ensure validity.

4.2 Intervention Protocols：

The experiment will be conducted at the university's golf training facility under the supervision of professional members of the research team, including the university team's head coach, team doctor, and a national-level golf referee. Data will be collected before and after the intervention, covering questionnaire assessments and golf skill tests (**Figure 2**). The baseline test and the formal test will be one week apart, and the experiment will take place on the same day of the week and at the same time (9:00–11:00 AM) to ensure sufficient recovery time and minimize the interference of residual effects (Fortes et al., 2022; Faro et al., 2023; Arenales Arauz et al., 2024).

Figure 2 Overview of the procedures within the experimental trials.

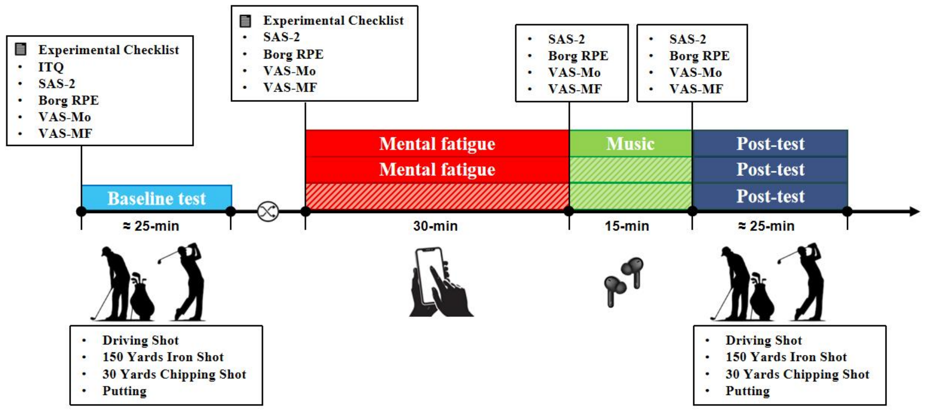

During the mental fatigue induction phase, a 30-minute Stroop task will artificially induce mental fatigue, simulating the mental fatigue state that athletes experience during competitions or training sessions (Jensen & Rohwer, 1966). The Stroop task is a classic cognitive control task widely used for inducing mental fatigue. Research in sports science has shown that a 30-minute Stroop task significantly increases subjective fatigue and reduces sports performance (Sun et al., 2021; Pan et al., 2024). This study will use a smartphone-based version of the Stroop task, where colour words (such as "red" and "blue") are displayed continuously, but the font colour does not match the word's meaning. Participants must ignore the word's meaning and respond only based on the font colour. The Stroop task requires sustained attention, conflict resolution, and cognitive inhibition, leading to cognitive resource depletion and mental fatigue accumulation. During the experiment, all participants will use their smartphones (with brightness and touch sensitivity standardised) and wear earphones to minimise external distractions. Participants in the MF-Mu and MF-nMu groups will undergo mental fatigue induction, while those in the CON group will only read a golf magazine in a relaxed manner.

During the music intervention phase, participants will listen to 15 minutes of classical music to facilitate mental fatigue recovery. Research has shown that short-term listening to classical music can effectively alleviate mental fatigue (Baghurst et al., 2014; Chen et al., 2009; Abbasi et al., 2024; Wang et al., 2025). Among classical music, Mozart's compositions are particularly effective in cognitive function recovery, attention enhancement, emotional regulation, and mental fatigue relief due to their bright and optimistic style, pure and elegant melodies, and harmonious rhythms (Pauwels et al., 2014; Colin et al., 2023; Waer et al., 2024). Therefore, This study selects Mozart's Eine Kleine Nachtmusik as the intervention music (Mozart et al., 1982), a piece that has been applied in previous mental fatigue recovery studies and has been validated as an effective intervention (Chen et al., 2009). The intervention duration is set at 15 minutes, as multiple studies have confirmed this duration to be sufficient to produce recovery effects without causing additional cognitive load or auditory fatigue (Chen et al., 2009; Axelsen et al., 2020; Jacquet et al., 2021). Based on research in the field of music therapy, the intervention volume should be maintained between 50–60 dBA.(Guerrero et al., 2012; Caparros-Gonzalez et al., 2018; Grafton-Clarke et al., 2019; Waer et al., 2024). The music will be played through a smartphone. Considering that the maximum volume of a smartphone is approximately 105–113 dBA (Kim & Han, 2018), the standardized intervention volume was

set at 50% of the maximum (52.5–56.5 dBA), with participants allowed to make slight adjustments to ensure listening comfort. Participants in the MF-Mu group will wear earphones and listen to the music for 15 minutes, while those in the MF-nMu and CON groups will wear earphones in the same environment but without audio, simply sitting at rest for 15 minutes. This experimental design effectively controls for non-musical factors, ensuring that the observed effects can be solely attributed to the music intervention.

5. Sample：

Participants in this study will be selected from athletes competing in the National College University Golf Championship (NCUGC) in China. The research team will collaborate with the head coaches of participating teams to identify universities willing to take part in the study. Subsequently, the head coaches and team doctors will conduct a preliminary screening of athletes based on the study's inclusion criteria, and the research team will verify the eligibility of the selected athletes before the experiment begins. The inclusion criteria are as follows: Aged between 18 and 24 years; Official participant in the NCUGC; A minimum of three years of golf-specific training experience; Trains at least five times per week. The exclusion criteria are: Color blindness; Hearing impairment; Insomnia; Physical injury; Mental health issues; Currently taking any medication.

The sample size for this study was calculated using G*Power 3.1.9.7 software (Faul et al., 2007), with the statistical analysis method set as ANOVA: Repeated measures, within-between interaction. The main input parameters included: a medium effect size of 0.25, a significance level of 0.05, a statistical power of 0.80, three groups, two measurement time points, a correlation of 0.5 between repeated measures, and a nonsphericity correction of 1. The calculation results indicated that 42 participants were required for this study. Considering a potential dropout rate of 15% (Cramer et al., 2016), a total of 48 golfers will be recruited.

6. Expected results：

According to the psychobiological model of athletic performance, psychological interventions that promote dopamine secretion, enhance motivation and attention, reduce perceived exertion, and alleviate mental fatigue are considered effective (Sun et al., 2022b). Studies have shown that dopamine-regulating psychological recovery interventions can effectively reduce mental fatigue and help maintain sport-specific performance in football and basketball athletes (Sun et al., 2022c; Cao et al., 2024). Music has been found to promote dopamine secretion (Stegemöller, 2014). Therefore, this study hypothesizes that music intervention is also applicable to golfers, helping to alleviate mental fatigue and effectively protect their sport-specific skills from the negative effects of mental fatigue.

Reference：

Abbasi, M., Esmaeili, R., Pourabdian, S., & Shakerian, M. (2024). Exploring the influence of music on cognitive performance in female assembly line workers at a medical device manufacturing unit. *Plos one*, *19*(10), e0309555. https://doi.org/10.1371/journal.pone.0309555

Angius, L., Merlini, M., Hopker, J., Bianchi, M., Fois, F., Piras, F., ... & Marcora, S. M. (2022). Physical and mental fatigue reduce psychomotor vigilance in professional football players. *International journal of sports physiology and performance*, *17*(9), 1391-1398. https://doi.org/10.1123/ijspp.2021-0387

Arenales Arauz, Y. L., Habay, J., Ocvirk, T., Mali, A., Russell, S., Marusic, U., ... & Roelands, B. (2024). The interplay of brain neurotransmission and mental fatigue: A research protocol. *Plos one*, *19*(9), e0310271. https://doi.org/10.1371/journal.pone.0310271

Axelsen, J. L., Kirk, U., & Staiano, W. (2020). On-the-spot binaural beats and mindfulness reduces the effect of mental fatigue. *Journal of Cognitive Enhancement*, *4*(1), 31-39. https://doi.org/10.1007/s41465-019-00162-3

Baghurst, T., Tapps, T., Boolani, A., Jacobson, B. H., & Gill, R. (2014). The influence of musical genres on putting accuracy in golf: An exploratory study. *J Athl Enhancement 3*, *5*(2). https://doi.org/10.4172/2324-9080.1000165

Broadie, M. (2012). Assessing golfer performance on the PGA TOUR. *Interfaces*, *42*(2), 146-165. https://doi.org/10.1287/inte.1120.0626

Brown, S., & Fry, J. (2022). Golf course condition and playability beyond the greens. *International Turfgrass Society Research Journal*, *14*(1), 40-46. 2022 Jun;14(1):40-6. https://doi.org/10.1002/its2.73

Campbell, M. J., & Moran, A. P. (2014). There is more to green reading than meets the eye! Exploring the gaze behaviours of expert golfers on a virtual golf putting task. *Cognitive processing*, *15*, 363-372. https://doi.org/10.1007/s10339-014-0608-2

Cao, S., Geok, S. K., Roslan, S., Sun, H., Lam, S. K., & Qian, S. (2022). Mental fatigue and basketball performance: a systematic review. *Frontiers in Psychology*, *12*, 819081. https://doi.org/10.3389/fpsyg.2021.819081

Cao, S., Liu, J., Geok, S. K., Sun, H., & Wang, X. (2024). Effects of brief mindfulness intervention on mental fatigue and recovery in basketball tactical performance. *PloS one*, *19*(12), e0306815. https://doi.org/10.1371/journal.pone.0306815

Caparros-Gonzalez, R. A., de la Torre-Luque, A., Diaz-Piedra, C., Vico, F. J., & Buela-Casal, G. (2018). Listening to relaxing music improves physiological responses in premature infants: a randomized controlled trial. *Advances in Neonatal Care*, *18*(1), 58-69. https://doi.org/10.1097/ANC.0000000000000448

Chan, A. W., Tetzlaff, J. M., Altman, D. G., Laupacis, A., Gøtzsche, P. C., Krleža-Jerić, K., ... & Moher, D. (2013a). SPIRIT 2013 statement: defining standard protocol items for clinical trials. *Annals of internal medicine*, *158*(3), 200-207. https://doi.org/10.7326/0003-4819-158-3-201302050-00583

Chan, A. W., Tetzlaff, J. M., Gøtzsche, P. C., Altman, D. G., Mann, H., Berlin, J. A., ... & Moher, D. (2013b). SPIRIT 2013 explanation and elaboration: guidance for protocols of clinical trials. *Bmj*, *346*. https://doi.org/10.1136/bmj.e7586

Chen, L., Sugi, T., Shirakawa, S., ZOU, J., & NAKAMURA, M. (2009). Neuro-physiological Features Related to Subjective Evaluation and Performance Assessment During Sustained Mental Calculation and Rest Break. *Transactions of Japanese Society for Medical and Biological Engineering*, *47*(2), 148-153. https://doi.org/10.11239/jsmbe.47.148

Colin, C., Prince, V., Bensoussan, J. L., & Picot, M. C. (2023). Music therapy for health workers to reduce stress, mental workload and anxiety: a systematic review. *Journal of Public Health*, *45*(3), e532-e541. https://doi.org/10.1093/pubmed/fdad059

Cosmo, B. G., de Menezes Vervloet, G., Rocha, J. P., Klein, M. P., dos Santos Silva, R., & Campos, V. R. (2022). Music therapy as a non-pharmacological intervention in Alzheimer's disease: an integrative review. *Rev Med (São Paulo)*, *101*(5), 197832. https://doi.org/10.11606/issn.1679-9836.v101i5e-197832

Cotterill, S. T., Sanders, R., & Collins, D. (2010). Developing effective pre-performance routines in golf: Why don't we ask the golfer?. *Journal of Applied Sport Psychology*, *22*(1), 51-64. https://doi.org/10.1080/10413200903403216

Cramer, H., Haller, H., Dobos, G., & Lauche, R. (2016). A systematic review and meta-analysis estimating the expected dropout rates in randomized controlled trials on yoga interventions. *Evidence-Based Complementary and Alternative Medicine*, *2016*(1), 5859729. https://doi.org/10.1155/2016/5859729

Davidow, D., Smith, M., Ross, T., James, G. L., Paul, L., Lambert, M., ... & Hendricks, S. (2023). Mental fatigue impairs tackling technique in amateur rugby union players. *International journal of sports physiology and performance*, *18*(9), 960-967. https://doi.org/10.1123/ijspp.2023-0159

Ding, C., Kim Geok, S., Sun, H., Roslan, S., Cao, S., & Zhao, Y. (2025). Does music counteract mental fatigue? A systematic review. *PloS one*, *20*(1), e0316252. https://doi.org/10.1371/journal.pone.0316252

Du, Y. J., Guan, Y. H., Thome, K. T., & Dong, J. C. (2024). Music therapy and music intervention for NSCLC patients undergoing PET with fear of cancer recurrence. *Integrative Cancer Therapies*, *23*, 15347354241269898. https://doi.org/10.1177/153473542412698

Englert, C., & Bertrams, A. G. (2016). Active relaxation counteracts the effects of ego depletion on performance under evaluative pressure in a state of ego depletion. *Sportwissenschaft*, *46*(2), 110-115. https://doi.org/110-115. 10.1007/s12662-015-0383-y

Faro, H., Fortes, L. D. S., Lima-Junior, D. D., Barbosa, B. T., Ferreira, M. E. C., & Almeida, S. S. (2023). Sport-based video game causes mental fatigue and impairs visuomotor skill in male basketball players. *International Journal of Sport and Exercise Psychology*, *21*(6), 1125-1139. https://doi.org/10.1080/1612197X.2022.2109187

Farrally, M. R., Cochran, A. J., Crews, D. J., Hurdzan, M. J., Price, R. J., Snow, J. T., & Thomas, P. R. (2003). Golf science research at the beginning of the twenty-first century. *Journal of sports sciences*, *21*(9), 753-765. https://doi.org/10.1080/0264041031000102123

Faul, F., Erdfelder, E., Lang, A. G., & Buchner, A. (2007). G* Power 3: A flexible statistical power analysis program for the social, behavioral, and biomedical sciences. *Behavior research methods*, *39*(2), 175-191. https://doi.org/10.3758/BF03193146

Finsterer, J., & Mahjoub, S. Z. (2014). Fatigue in healthy and diseased individuals. *American Journal of Hospice and Palliative Medicine®*, *31*(5), 562-575. https://doi.org/10.1177/1049909113494748

Fortes, L. S., Lima-Junior, D., Barbosa, B. T., Faro, H. K., Ferreira, M. E., & Almeida, S. S. (2022). Effect of mental fatigue on decision-making skill and visual search behaviour in basketball players: an experimental and randomised study. *International Journal of Sport and Exercise Psychology*, 1-20. https://doi.org/10.1080/1612197X.2022.2058055

Fouillouze, A., Lacoeuilhe, A., & Truong, M. X. A. (2023). A step towards a greener green? Investigating golfers' relationships with nature and attitudes about biodiversity conservation in golf courses. *Journal of Outdoor Recreation and Tourism*, *43*, 100659. https://doi.org/10.1016/j.jort.2023.100659

Ghetti, C. M. (2012). Music therapy as procedural support for invasive medical procedures: Toward the development of music therapy theory. *Nordic Journal of Music Therapy*, *21*(1), 3-35. https://doi.org/10.1080/08098131.2011.571278

GIACOBBI, P., Foore, B., & Weinberg, R. S. (2004). Broken clubs and expletives: The sources of stress and coping responses of skilled and moderately skilled golfers. *Journal of applied sport psychology*, *16*(2), 166-182. https://doi.org/10.1080/10413200490437688

Grafton-Clarke, C., Grace, L., & Harky, A. (2019). Music therapy following cardiac surgery—is it an effective method to reduce pain and anxiety?. *Interactive cardiovascular and thoracic surgery*, *28*(5), 722-727. https://doi.org/10.1093/icvts/ivy311

Graves, R. M., & Cornish, G. S. (1998). *Golf course design*. John Wiley & Sons.

Guerrero, J. M., Castaño, P. M., Schmidt, E. O., Rosario, L., & Westhoff, C. L. (2012). Music as an auxiliary analgesic during first trimester surgical abortion: a randomized controlled trial. *Contraception*, *86*(2), 157-162. https://doi.org/10.1016/j.contraception.2011.11.017

Habay, J., Van Cutsem, J., Verschueren, J., De Bock, S., Proost, M., De Wachter, J., ... & Roelands, B. (2021). Mental fatigue and sport-specific psychomotor performance: a systematic review. *Sports Medicine*, *51*, 1527-1548. https://doi.org/10.1007/s40279-021-01429-6

Han, J., Xie, H., Cong, S., Wang, M., Ni, S., Wu, Y., & Zhang, A. (2024). Effectiveness of smartphone-based music intervention on perinatal depression: protocol for a randomized controlled trial. *BMC psychology*, *12*(1), 1-6. https://doi.org/10.1186/s40359-024-02141-6

Hasegawa, Y., Okada, A., & Fujii, K. (2021). Skill differences in a discrete motor task emerging from the environmental perception phase. *Frontiers in Psychology*, *12*, 697914. https://doi.org/10.3389/fpsyg.2021.697914

Jacquet, T., Poulin-Charronnat, B., Bard, P., Perra, J., & Lepers, R. (2021). Physical activity and music to counteract mental fatigue. *Neuroscience*, *478*, 75-88. https://doi.org/10.1016/j.neuroscience.2021.09.019

Jensen, A. R., & Rohwer Jr, W. D. (1966). The Stroop color-word test: a review. *Acta psychologica*, *25*, 36-93. https://doi.org/10.1016/0001-6918(66)90004-7

Kim, G., & Han, W. (2018). Sound pressure levels generated at risk volume steps of portable listening devices: types of smartphone and genres of music. *BMC Public Health*, *18*, 1-7. https://doi.org/10.1186/s12889-018-5399-4

Lorek, M., Bąk, D., Kwiecień-Jaguś, K., & Mędrzycka-Dąbrowska, W. (2023, June). The effect of music as a non-pharmacological intervention on the physiological, psychological, and social response of patients in an intensive care unit. In *Healthcare* (Vol. 11, No. 12, p. 1687). MDPI. https://doi.org/10.3390/healthcare11121687

Magee, W. L. (2005). Music therapy with patients in low awareness states: approaches to assessment and treatment in multidisciplinary care. *Neuropsychological Rehabilitation*, *15*(3-4), 522-536. https://doi.org/10.1080/09602010443000461

Martin, K., Meeusen, R., Thompson, K. G., Keegan, R., & Rattray, B. (2018). Mental fatigue impairs endurance performance: a physiological explanation. *Sports medicine*, *48*(9), 2041-2051. https://doi.org/10.1007/s40279-018-0946-9

Mizuno, K., Tanaka, M., Yamaguti, K., Kajimoto, O., Kuratsune, H., & Watanabe, Y. (2011). Mental fatigue caused by prolonged cognitive load associated with sympathetic hyperactivity. *Behavioral and brain functions*, *7*, 1-7. https://doi.org/10.1186/1744-9081-7-17

Mozart, W. A., Brainin, N., Klier, M., Lovett, M., Nissel, S., Schidlof, P., ... & Quartet, A. S. (1982). *Eine kleine Nachtmusik G dur KV 525 Ein musikalischer Spass KV 522" Dorfmusikanten-Sextett"*. Deutsche Grammophon.

Pan, X., Soh, K. G., Jaafar, W. M. W., Soh, K. L., Deng, N., Cao, S., ... & Liu, H. (2025). Mental fatigue in golf: A systematic review. *PloS one*, *20*(2), e0310403. https://doi.org/10.1371/journal.pone.0310403

Pan, X., Soh, K. G., & Soh, K. L. (2024). Viable strategies for enhancing performance in ball sports by mitigating mental fatigue: A systematic review. *PloS one*, *19*(11), e0313105. https://doi.org/10.1371/journal.pone.0313105

Pauwels, E. K., Volterrani, D., Mariani, G., & Kostkiewics, M. (2014). Mozart, music and medicine. *Medical Principles and Practice*, *23*(5), 403-412. https://doi.org/10.1159/000364873

Pilgrim, J., Kremer, P., & Robertson, S. (2018). The development of a tournament preparation framework for competitive golf: A Delphi study. *European Journal of Sport Science*, *18*(7), 930-939. https://doi.org/10.1080/17461391.2018.1469673

Pilgrim, J., Robertson, S., & Kremer, P. (2016). A qualitative investigation into the role of the caddie in elite-level golf. *International Journal of Sports Science & Coaching*, *11*(4), 599-609. https://doi.org/10.1177/1747954116654783

Proost, M., Habay, J., De Wachter, J., De Pauw, K., Rattray, B., Meeusen, R., ... & Van Cutsem, J. (2022). How to tackle mental fatigue: a systematic review of potential countermeasures and their underlying mechanisms. *Sports Medicine*, *52*(9), 2129-2158. https://doi.org/10.1007/s40279-022-01678-z

Qi, P., Ru, H., Gao, L., Zhang, X., Zhou, T., Tian, Y., ... & Sun, Y. (2019). Neural mechanisms of mental fatigue revisited: New insights from the brain connectome. *Engineering*, *5*(2), 276-286. https://doi.org/10.1016/j.eng.2018.11.025

Shaw, M., Birch, P. D., & Runswick, O. R. (2021). An in-situ examination of cognitive processes in professional and amateur golfers during green reading. *Sport, Exercise, and Performance Psychology*, *10*(2), 273. https://doi.org/10.1037/spy0000261

Smith, M. R., Coutts, A. J., Merlini, M., Deprez, D., Lenoir, M., & Marcora, S. M. (2016). Mental fatigue impairs soccer-specific physical and technical performance. *Medicine and science in sports and exercise*, *48*(2), 267-276. https://doi.org/10.1249/MSS.0000000000000762

Stegemöller, E. L. (2014). Exploring a neuroplasticity model of music therapy. *Journal of Music Therapy*, *51*(3), 211-227. https://doi.org/10.1093/jmt/thu023

Sun, H., Soh, K. G., Roslan, S., Wazir, M. R. W. N., & Soh, K. L. (2021). Does mental fatigue affect skilled performance in athletes? A systematic review. *PloS one*, *16*(10), e0258307. https://doi.org/10.1371/journal.pone.0258307

Sun, H., Soh, K. G., Mohammadi, A., Wang, X., Bin, Z., & Zhao, Z. (2022a). Effects of mental fatigue on technical performance in soccer players: A systematic review with a meta-analysis. *Frontiers in public health*, *10*, 922630. https://doi.org/10.3389/fpubh.2022.922630

Sun, H., Soh, K. G., Roslan, S., Wazir, M. R. W. N., Mohammadi, A., Ding, C., & Zhao, Z. (2022b). Nature exposure might be the intervention to improve the self-regulation and skilled performance in mentally fatigue athletes: A narrative review and conceptual framework. *Frontiers in Psychology*, *13*, 941299. https://doi.org/10.3389/fpsyg.2022.941299

Sun, H., Soh, K. G., & Xu, X. (2022c). Nature scenes counter mental fatigue-induced performance decrements in soccer decision-making. *Frontiers in Psychology*, *13*, 877844. https://doi.org/10.3389/fpsyg.2022.877844

Tanaka, M., Ishii, A., & Watanabe, Y. (2014). Neural effects of mental fatigue caused by continuous attention load: a magnetoencephalography study. *Brain research*, *1561*, 60-66. https://doi.org/10.1016/j.brainres.2014.03.009

Van Cutsem, J., Marcora, S., De Pauw, K., Bailey, S., Meeusen, R., & Roelands, B. (2017). The effects of mental fatigue on physical performance: a systematic review. *Sports medicine*, *47*, 1569-1588. https://doi.org/10.1007/s40279-016-0672-0

Veness, D., Patterson, S. D., Jeffries, O., & Waldron, M. (2017). The effects of mental fatigue on cricket-relevant performance among elite players. *Journal of sports sciences*, *35*(24), 2461-2467. https://doi.org/10.1080/02640414.2016.1273540

Waer, F. B., Alexe, D. I., Alexe, C. I., Eken, Ö., Păun, L. I., & Sahli, S. (2024). Impact of classical music listening on cognitive and functional performances in Middle-aged women. *Applied Sciences*, *14*(15), 6779. https://doi.org/10.3390/app14156779

Wang, H. T., Chen, Y. S., Rekik, G., Yang, C. C., Lai, M. S., & Tai, H. L. (2022). The effect of listening to preferred music after a stressful task on performance and psychophysiological responses in collegiate golfers. *PeerJ*, *10*, e13557. https://doi.org/10.7717/peerj.13557

Wang, H. T., Tai, H. L., Yang, C. C., & Chen, Y. S. (2020). Acute effects of self-selected music intervention on golf performance and anxiety level in collegiate golfers: A crossover study. *International Journal of Environmental Research and Public Health*, *17*(20), 7478. https://doi.org/10.3390/ijerph17207478

Wang, Z., Xu, W., Zhang, C., Zhang, C., Liu, Y., Chen, P., ... & Wang, L. (2025). Music boosts the recovery of attention after mental fatigue in healthy young male subjects: A human auditory event-related potential study. *Behavioural Brain Research*, *485*, 115539. https://doi.org/10.1016/j.bbr.2025.115539
